# Supplementary material for: IL-9 aggravates SARS-CoV-2 infection and exacerbates associated airway inflammation
Source: Nat Commun. 2023 Jul 10;14:4060. doi: 10.1038/s41467-023-39815-5 (PMC10333319; doi:10.1038/s41467-023-39815-5)
Supplement: Supplementary file 1 — Supplementary Information [file 41467_2023_39815_MOESM1_ESM.pdf]

Supplementary Information

IL-9 aggravates SARS-CoV-2 infection and exacerbates associated airway inflammation

Srikanth Sadhu[1,2], Rajdeep Dalal[1], Jyotsna Dandotiya[1], Akshay Binayke[1], Virendra Singh[1], Manas Ranjan Tripathy[1,2], Vinayaka Das[1], Sandeep Goswami[1], Shakti Kumar[3], Zaigham Abbas Rizvi[1,2], Amit Awasthi[#1,2]

Supplementary Fig. 1

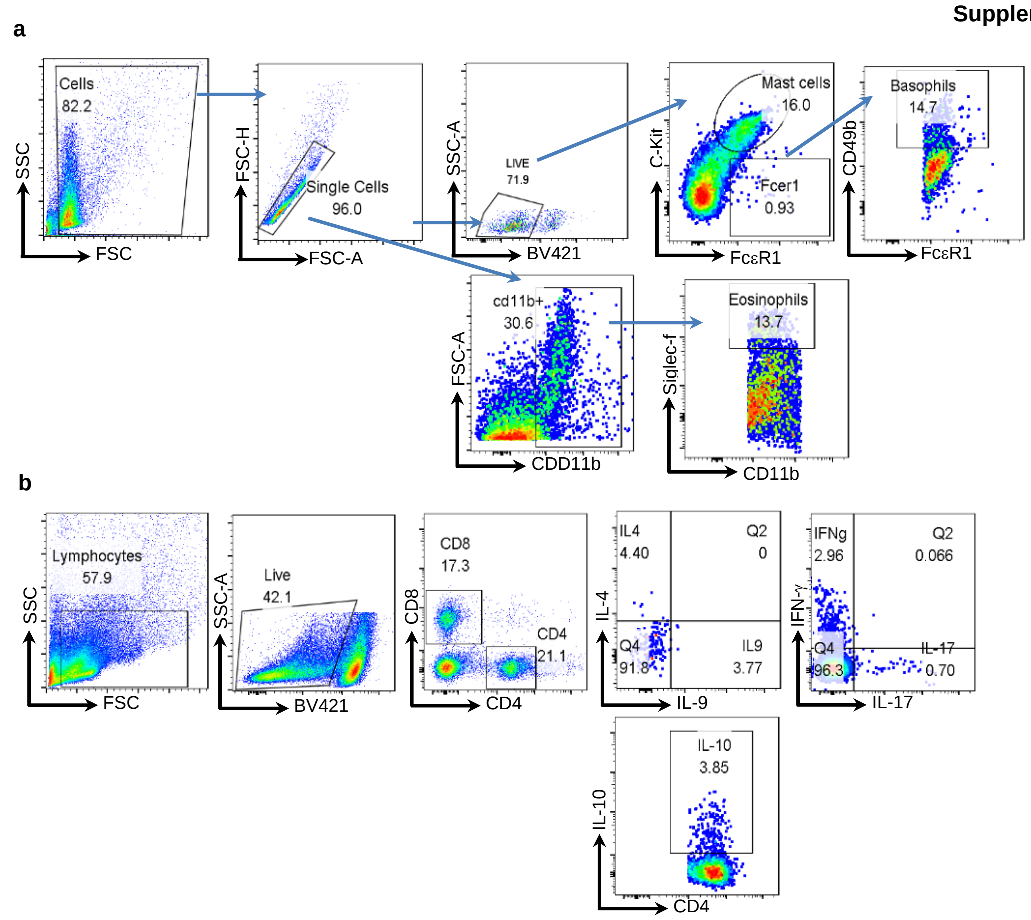

Supplementary Fig.1. Gating strategy of BALF mast cells, eosinophils, basophils and intra cellular cytokine staining

a) Mice BALF cells were collected and analyzed using flow cytometry. Initially, cells were gated based on forward scatter (FSC-A) versus side scatter (SSC-A), and singlets were selected using FSC-H versus FSC-Afollowed by selecting live cells using Live/Dead staining. Mast cells were identified by gating on double-positive cells using Fcεr1 and C-Kit surface markers while basophils were identified by surface staining of Fcεr1 and CD49b. Eosinophils were identified as CD11b and Siglec-f positive cells. The similar gating strategy was consistently applied throughout the manuscript wherever these cell types were analyzed by flow cytometry. **b)** The gating strategy used in the analysis of intracellular cytokines in BALF, spleen, and dLN. Cells were initially gated based on FSC-A versus SSC-A, and singlets were selected using FSC-H versus FSC-A. Singlets were then gated based on Live/Dead staining to identify live cells. The live cells were further gated based on the presence of CD4 and CD8 markers. The CD4 cells were subsequently gated for the presence of IL-9, IL-4, IFN-γ, IL-10, and IL-17.

Supplementary Fig. 2

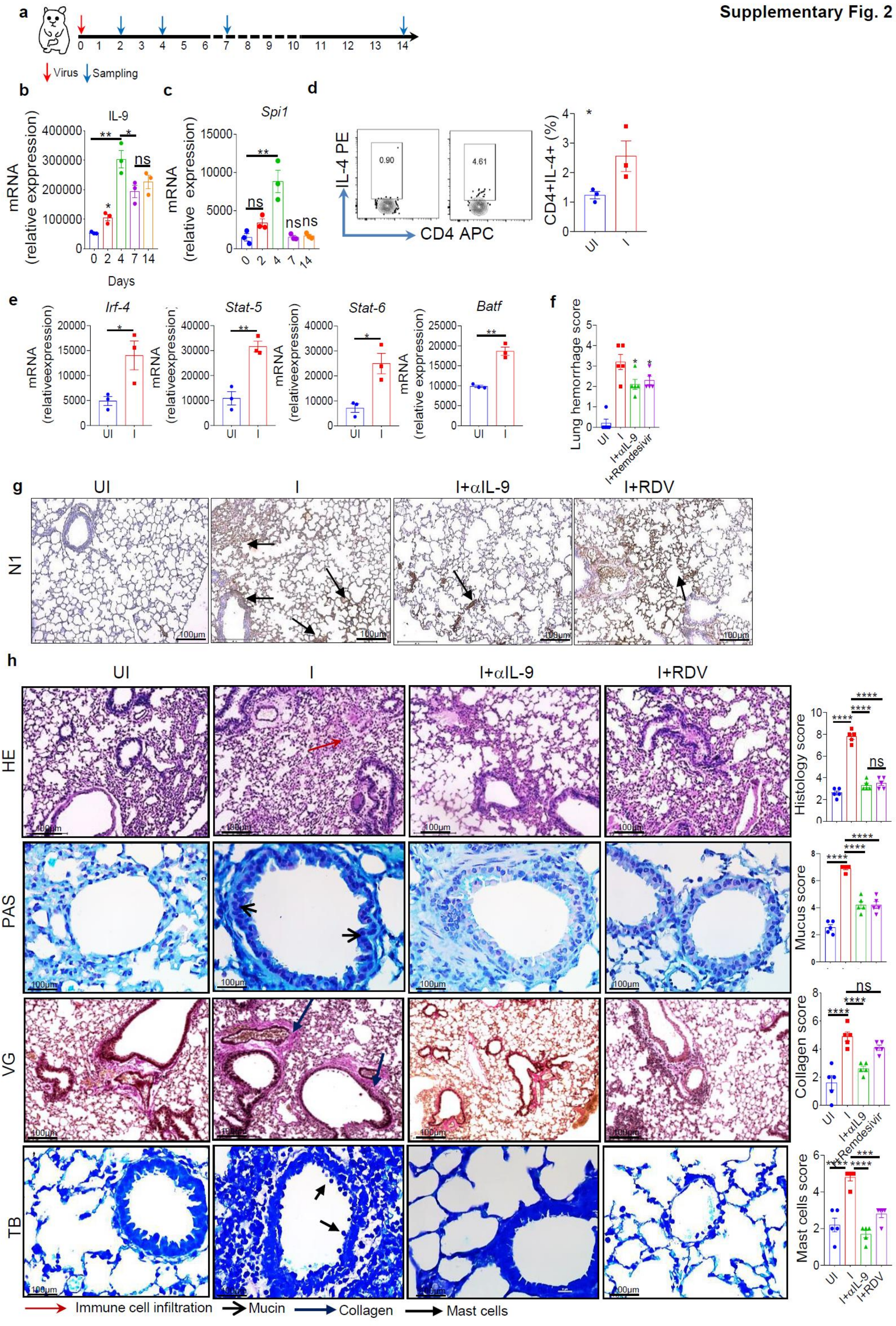

Supplementary Fig. 2: Effect of IL-9 on SARS-CoV-2 infection in hamsterandACE2.Tg mice a) Schematic representation indicating infection in hamster and collection of samples. **b, c)** Total RNA was isolated from SARS-CoV-2-infected lung tissues of hamster (n=3) of 0, 2, 4, 7 and 14 days post infection, and gene expression was performed for *Il-9* and PU.1, *p<0.05, **p<0.005 (one way ANOVA followed by Tukey's multiple comparison test); Bar graph represents as a mean +SEM. **d)** FACS dot plot represents percentage of $CD4^{+}IL\text{-}4^{+}$ T cells and bargraph represents +SEM; n=3 mice per group; p<0.05. **e)** mRNA expression for *Irf4*, *Stat5*, *Stat6*, and *Batf* in uninfected, SARS-CoV-2-infected mice lung samples, n=3 mice per group; bar graph represents as a mean +SEM *P= 0.0425, **P=0.0028 (one way ANOVA, followed by Tukey's multiple comparison test. **f)** Lung haemorrhage scored on a scale of 0–5, where 0 is a normal pink healthy lung and 5 is a completely dark red lung and lesions present on lungs at 7 dpi, (n = 5) (*P < 0.0430; One-way ANOVA and Tukey's multiple comparison test); bar graph represents as a mean +SEM. **g)** Representative images of immunostaining for SARS-CoV-2-N antigen (brown; 40x magnification; 100 µm scale bar) in lung tissue sections at 7 dpi; (n=4 mice per group; experiment was performed once). **h)** Images of H&E, PAS (Periodic acid Schiff), VG (Van Gieson) and TB (Toludine blue) staining shows, infiltration of immune cells & lung injury, mucin secretion, collagen thickening and mast cells presence respectively (h) HE&VG: 20x magnification, 100µm scale bar; PAS&TB: 60X magnification, 50µm scale bar; (b). (n=5 mice per group; experiment was performed once) Bar graph represents as a mean +SEM; one-way ANOVA followed by Tukey's multiple comparison test. (***P=0.0002, ****P<0.0001; one-way ANOVA).

Supplementary Fig. 3

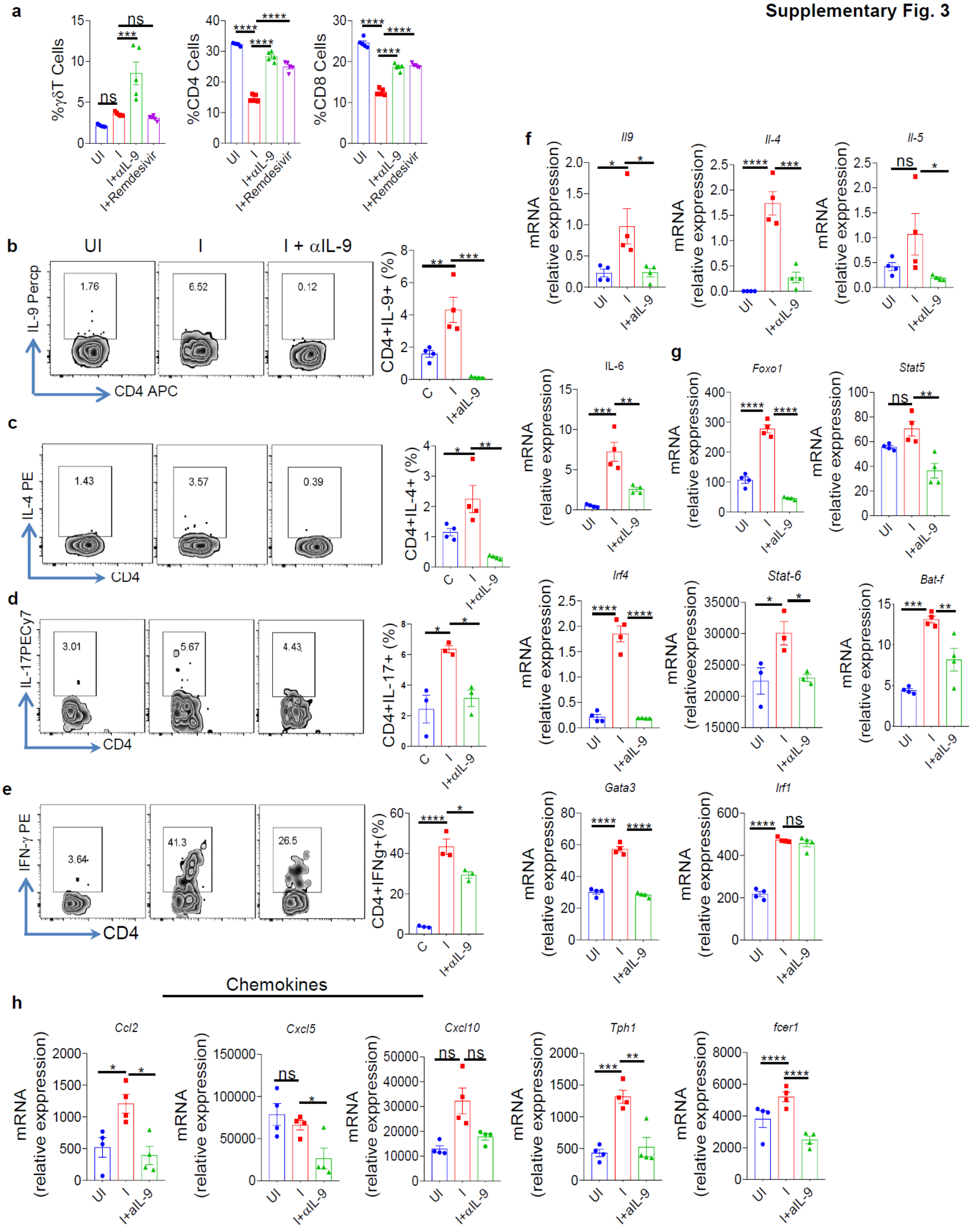

Supplementary Fig. 3: Effect of anti-IL-9 treatment on SARS-CoV-2 infection in ACE2.Tg mice

a) Frequency of γδ^{+}, CD4^{+} and CD8^{+} T cell was determined in BAL; bar graph represents as a mean ± SEM (n=5 mice per group) (*p<0.05, **p<0.005, ****p<0.0001, ns= non-significant; two-way ANOVA followed by Tukeys multiple comparison test). **b-e)** Effect of anti-IL-9 treatment on IL-9, IL-4, IL-17 and IFN-γ was determined by intracellular cytokine staining, data representative of five individual mice (n=5 mice per group), Bar represents as

a mean ± SEM; *p<0.05, ***p<0.0005, ****p<0.0001(one way ANOVA followed by Tukey's multiple comparison test). **f)** mRNA expression of Il9, Il4, Il5 and Il6 measured in BAL samples by qPCR (n=4, *p=0.0421, **p=0.0098, ***p=0.0002, ****p<0.0001. one way ANOVA, followed by Tukeys multiple comparison rest); Bar graph represents as a mean ± SEM. **g)** mRNA expression of *Foxo1, Stat5, Irf4, Stat6, Batf, Gata3* and *Irf1* genes determined by qPCR in SARS-CoV-2-infected BAL samples of ACE2.Tg mice (n=4 mice per group; bar graph represents as a mean ± SEM).One-way ANOVA followed by Tukey's multiple comparison test); *p<0.05, **p<0.005, ***p<0.0005, ****p<0.0001, ns= non-significant. **h)** Relative mRNA expression of chemokines (*Ccl2, Cxcl5, Cxcl10, Tph1* and *Fcεr1*). Significance difference: **p < 0.01, ***p< 0.001, ****p<0.0001, ns= non-significant (one-way analysis followed by Tukey's multiple comparison test); Bar graph represents as a mean ± SEM (n=4).

Supplementary Fig. 4

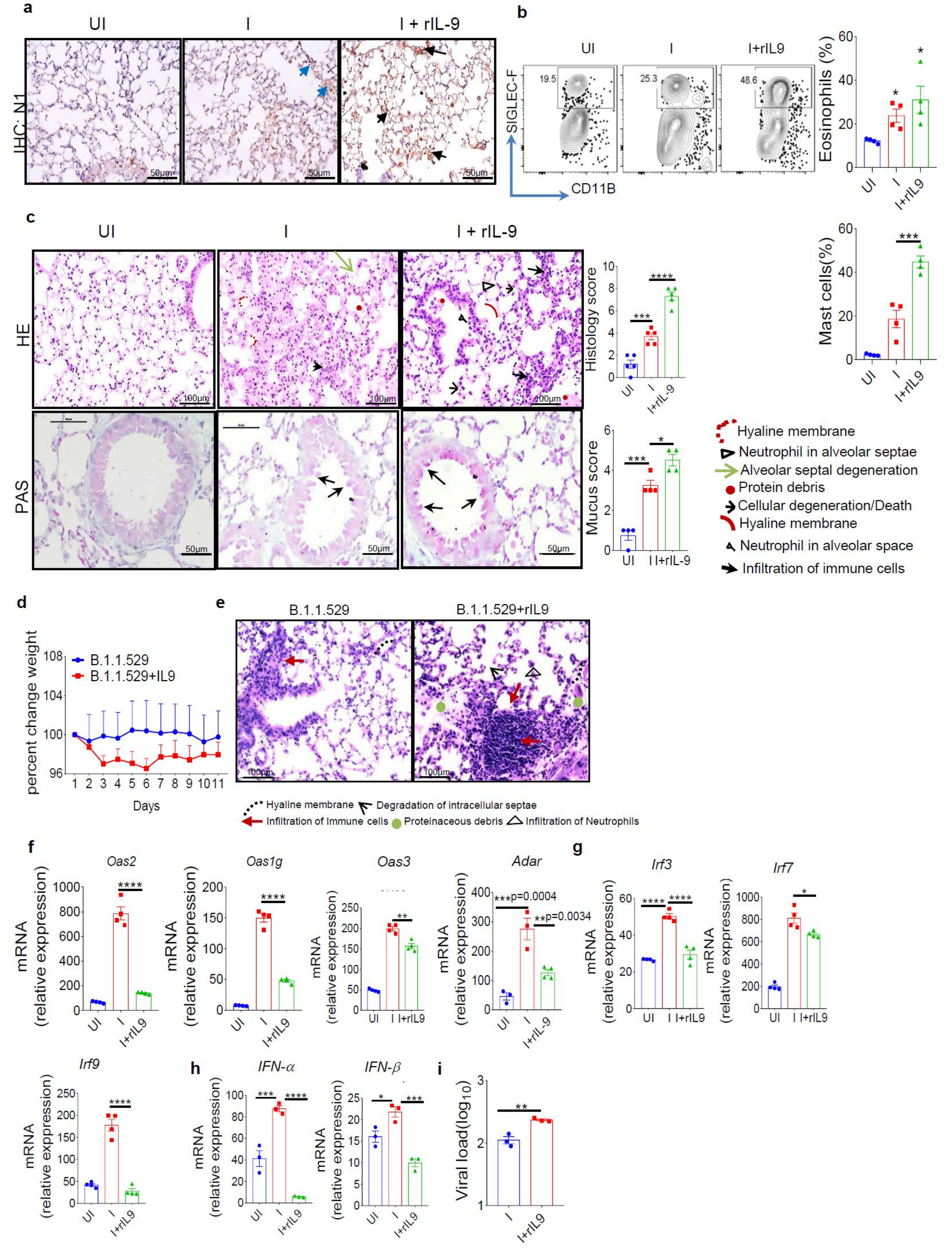

Supplementary Fig. 4: Exogenous IL-9 enhanced SARS-CoV-2 Infection and lung inflammation.

a) Immunostaining for SARS-CoV-N antigen (brown) in lung tissue sections at 7 dpi; (n=5 mice per group; experiment was performed once; 100μm scale bar). **b**) Eosinophil percentage

was determined in BALF (n=4 mice per group).; *p=0.0216, one-way ANOVA followed by Tukey's multiple comparison test. Bar graph represents as a mean ±SEM. **c)** Representative images shows the HE (100μm scale bar), and PAS (periodic acid-Schiff; 50μm scale bar) staining and bar graph represents histological score for various parameters (60 x magnifications); (n=5 mice per group)*p<0.05, ***p<0.02, ****p<0.0001. Bar graph represents as a mean ±SEM; one-way ANOVA followed by Tukey's multiple comparison test. **d)** Percentage change in body weight of ACE2.Tg mice infected with B.1.1.529 with or without rIL-9 treatment (n=5 mice per group); two-way ANOVA; Bar graph represents as a mean ±SEM. **e)** Image shows the H&E staining of B.1.1.529 infected lung samples (n=5 mice per group;100μm scale bar). **f-h)** Relative mRNA expression of *Oas1g, Oas2, Oas3 Adar*, and transcription factors *Irf3, Irf7, Irf9* in lung tissues.(*p<0.05, **p<0.005, ***p<0.0005, ****p<0.0001, one-way ANOVA followed by Tukey's multiple comparison test); (n=4 mice per group); Bar graph represents as a mean ±SEM. **i)** A549 cells were infected (0.03MOI) and cultured in the presence or absence of rIL9. Viral load was measured by qPCR; (n=3 biological replicates); bar graph represents as a mean ±SEM (students t test) **p<0.005.

Supplementary Fig. 5

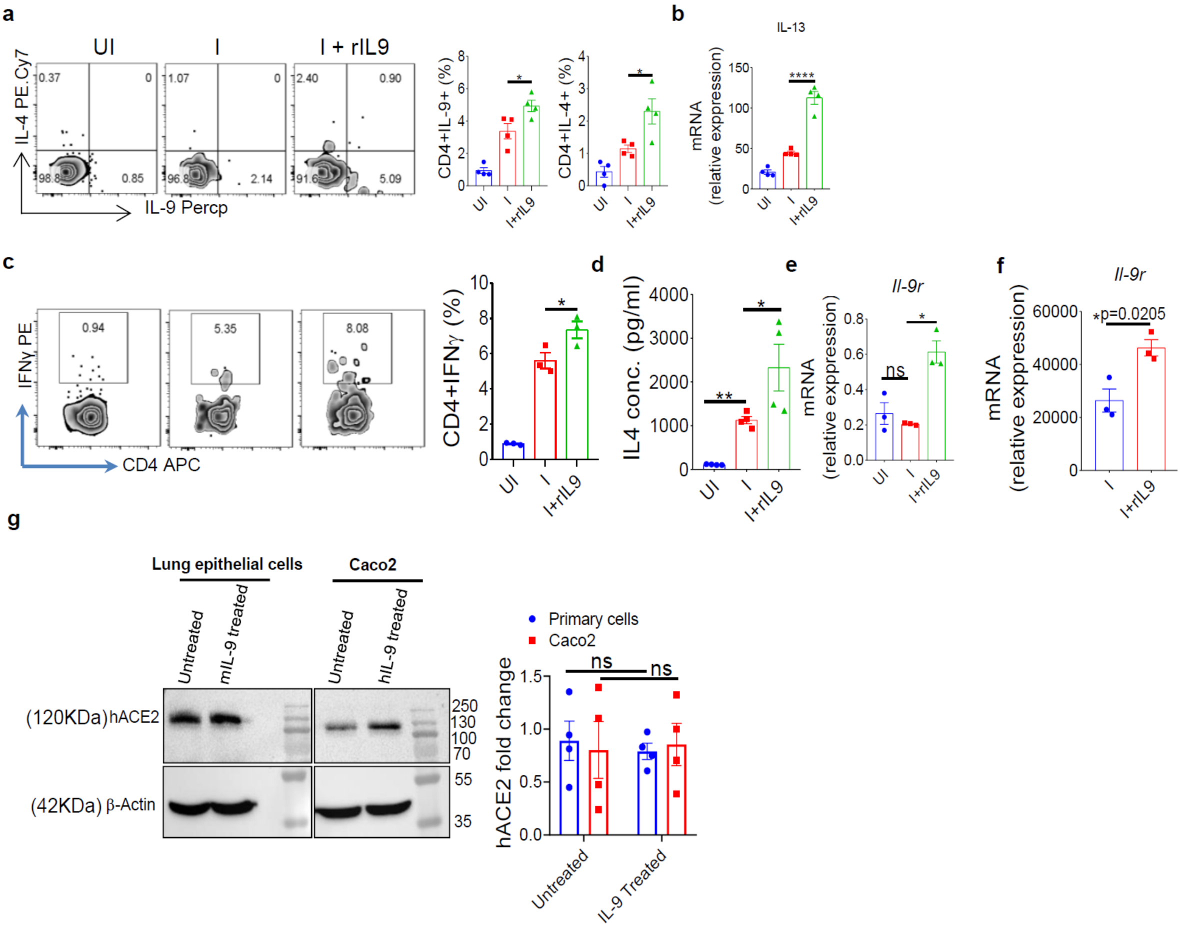

Supplementary Fig. 5: Effect of exogenous IL-9 on SARS-CoV-2 infection *in vitro* and *in vivo*.

a, c) IL-9, IL-4 (n=4 mice per group) and IFN-γ (n=3 mice per group) induction in BAL samples in CD4$^+$ T cells was determined by FACS. *p=0.0255, *p<0.0415, One-way ANOVA followed by Tukey's multiple comparison test; Bar graph represents as a mean ± SEM. mRNA expression of IL-13 by qPCR (****p<0.0001) (**b**), and (**d**) IL-4 quantification in BAL was estimated by ELISA (n=4 mice per group); bar graph represents ± SEM (one-way ANOVA followed by Tukey's multiple comparison test) *p=0.0484, **p=0.0075. hACE2 intestinal epithelial cells were isolated and infected with SARS-CoV-2 in the presence or absence of rIL-9, cells were lysed in Trizol and used for mRNA expression of *Il9*r by qPCR (**e**), in vivo (**f**) (n=3mice per group). **g)** Western blot analysis of mice lung epithelial cells and Caco2 cells shows that rIL9 doesn't influence the hACE2 expression; Bar graph represents four individual experiments (n=4); ns= non-significant. (*p<0.01, one way ANOVA followed by Tukey's multiple comparison test).

Supplementary Fig. 6

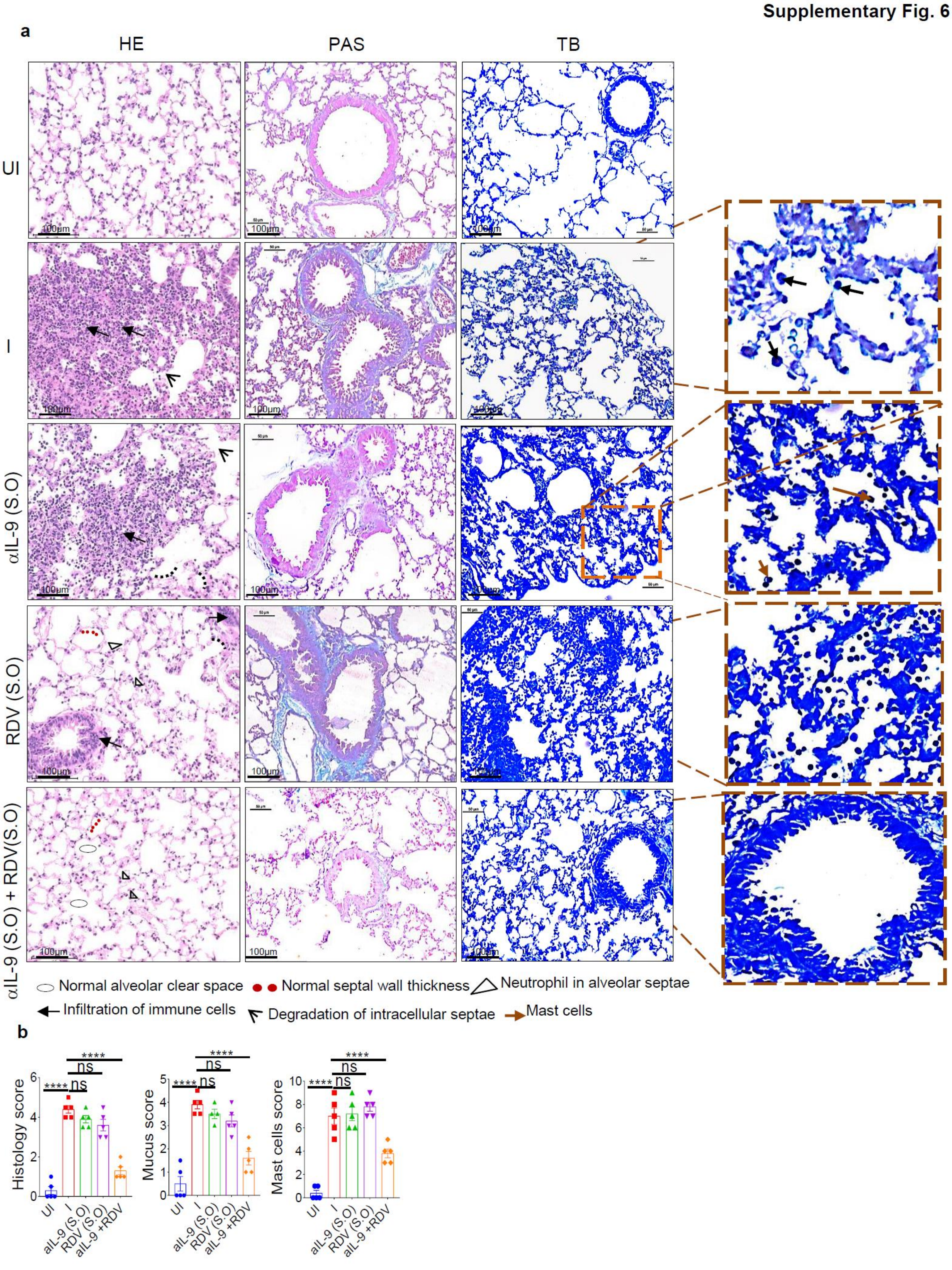

Supplementary Fig. 6: Histopathological analysis of SARS-CoV-2-infected lungs of combinatorial treatment of anti-IL-9 antibody with RDV

a) Gating strategy shows the mast cells, basophils and eosinophils in lungs. **b)** Representative histological lung images were shown for mock-infected or administered RDV (S.D), anti-IL-9 antibody (S.D), or RDV plus anti-IL-9 antibody. Symbols identifying example features of

the infection are indicated in the Fig (HE: 100µm scale bar; PAS&TB: 50µm);(n=5 mice per group); bar graph represents as a mean +SEM; one-way ANOVA followed by Tukey's multiple comparison test. All images were taken at the same magnification. ****p<0.0001, ns= non-significant.

Supplementary Fig. 7

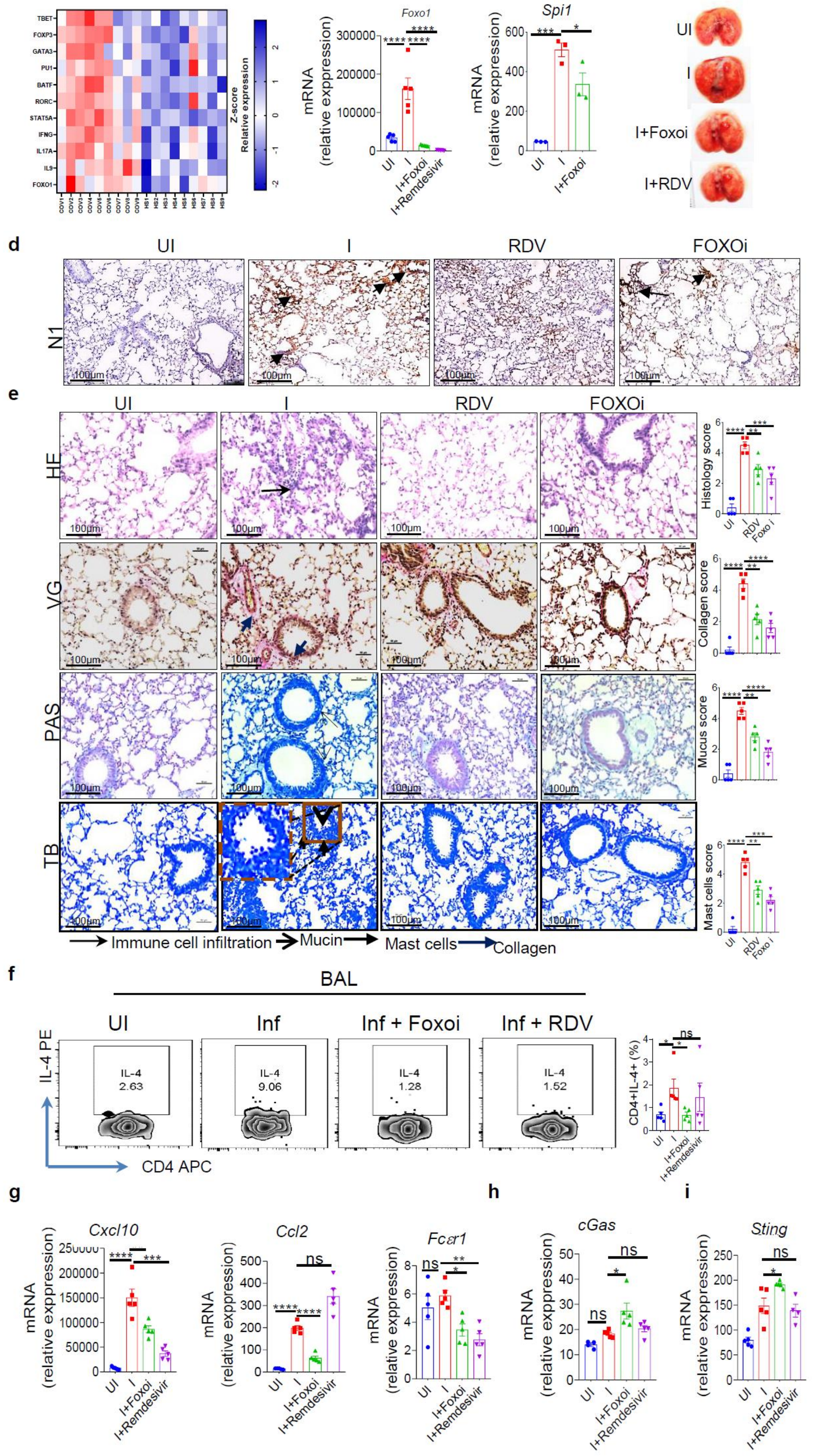

Supplementary Fig. 7: Foxo1 inhibition makes ACE2.Tg mice resistance to SARS-CoV-2 infection. a) The median of Z-scores of log2 transformed relative gene expression of symptomatic COVID-19 patients and healthy participants (n=9 each) is represented in the form of heatmap. b) Relative Foxo1 and Pu.1 mRNA expression levels were measured by

qRT-PCR (one-way ANOVA followed by Tukey’s test); (n=5 mice per group) *p<0.05, ***p<0.0005,****p<0.0001; Bar graph represents as a mean +SEM. c) Gross morphological changes and Lung haemorrhage scored on 7 dpi. d) IHC for the SARS-CoV-2-N protein antigen; (n=5 mice per group; experiment was performed once); 100μm scale bar. e) Images of H&E, VG, PAS and TB staining respectively. Bar shows as a mean ±SEM. (**P=0.0019, ***P=0.0005, ****P<0.0001; one-way ANOVA followed by Tukey’s multiple comparison test) (n=5 mice per group); HE,VG,PAS: 100μm scale bar; TB: 100μm scale bar. f) FACS plot represents IL-4^{+}CD4^{+} T cell frequency. n=5 mice, *p=0.0486 one-way ANOVA, followed by Tukey's multiple comparison test; Bar graph represents as a mean +SEM. g) Relative mRNA levels of *CxcL10, Ccl2,* and *Fcεr1* measured by RT-PCR (One way ANOVA followed by Tukey’s multiple comparison test); (n=4 mice per group);*p=0.0508, **p<0.0079, ***p=0.0008, ****p<0.0001, ns= non-significant; Bar graph represents as a mean ±SEM. h-i) Relative mRNA expression of cGAS and STING; *p<0.05, ns= non-significant; bar graph represents ±SEM; One-way ANOVA followed by Tukey’s multiple comparison test (n=4 mice per group).

Supplementary Fig. 8

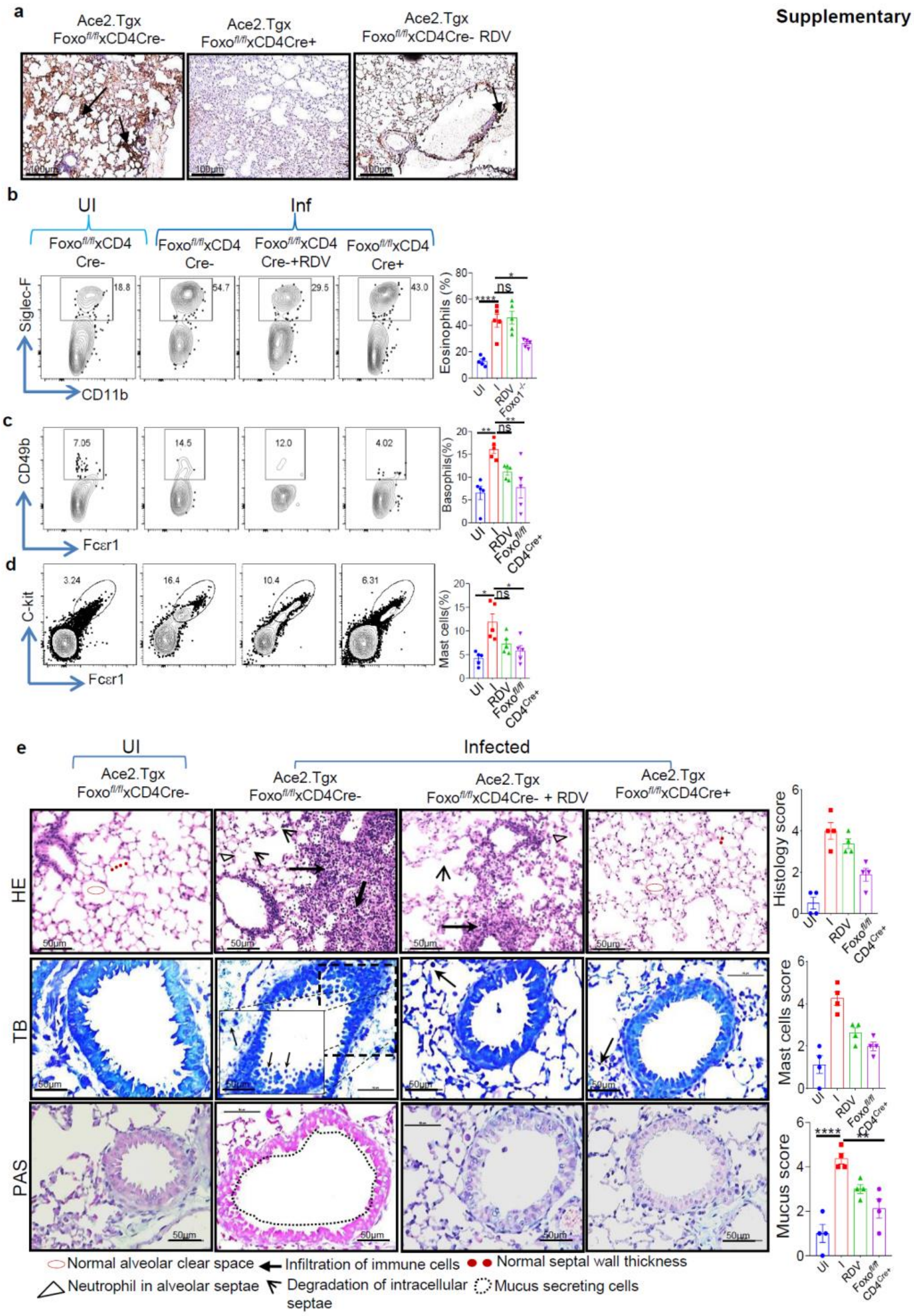

Supplementary Fig. 8: Foxo1 exacerbates SARS-CoV-2 infection. a) Representative images shows SARS-CoV-N antigen (brown) and haematoxylin stained nuclei (blue) in mouse lung tissue sections 7 dpi; (n=5 mice per group; experiment was performed once; 100µm). **b-d**) Eosinophils, mast cells, and basophils percentage population were analysed by FACS; *p=0.0180, **p=0.0076 (one way ANOVA followed by Tukey's multiple comparison test); (n=5 mice per group); Bar graph represents as a mean +SEM. **e**) Three randomly chosen high power (HE: 100µm; TB&PAS: 50µm scale bar) fields of different groups of lungs were assessed per mouse. Symbols identifying example features of disease are indicated in the Fig. *p<0.05, **p<0.005, ****p<0.0001; Bar graph represents as a mean +SEM (n=4 mice per group); one-way ANOVA; Tukey's multiple comparison test.

Supplementary Fig. 9

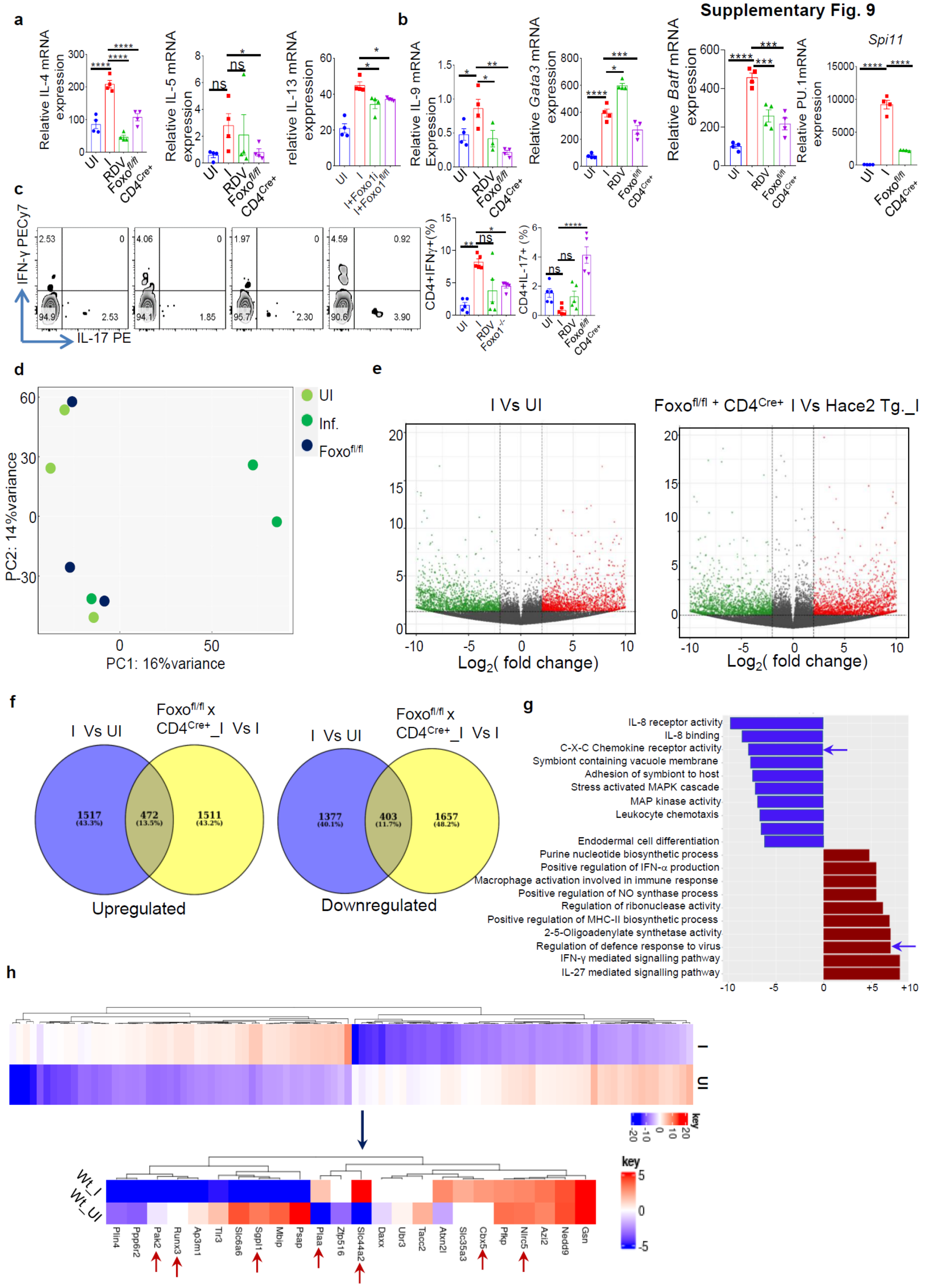

Supplementary Fig. 9 Cont

i

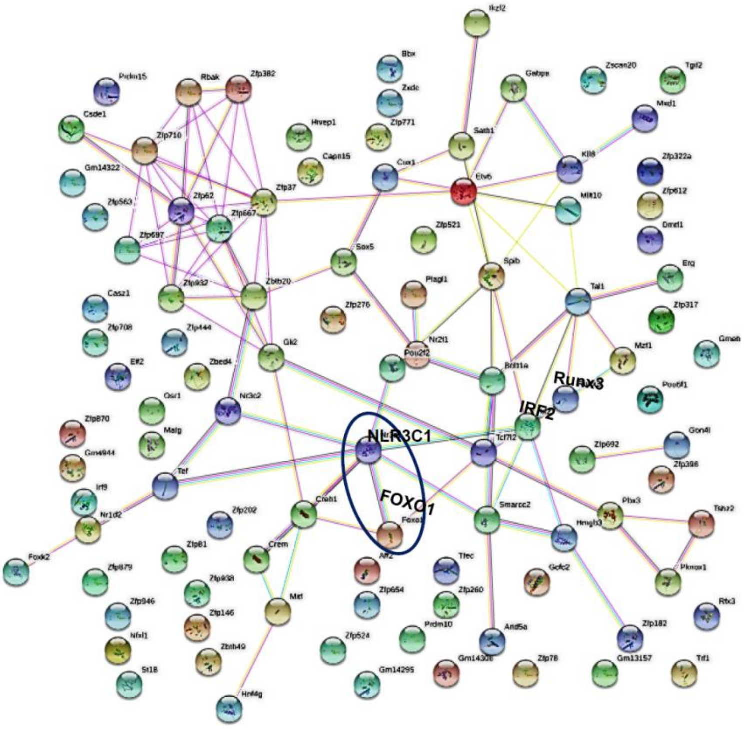

j

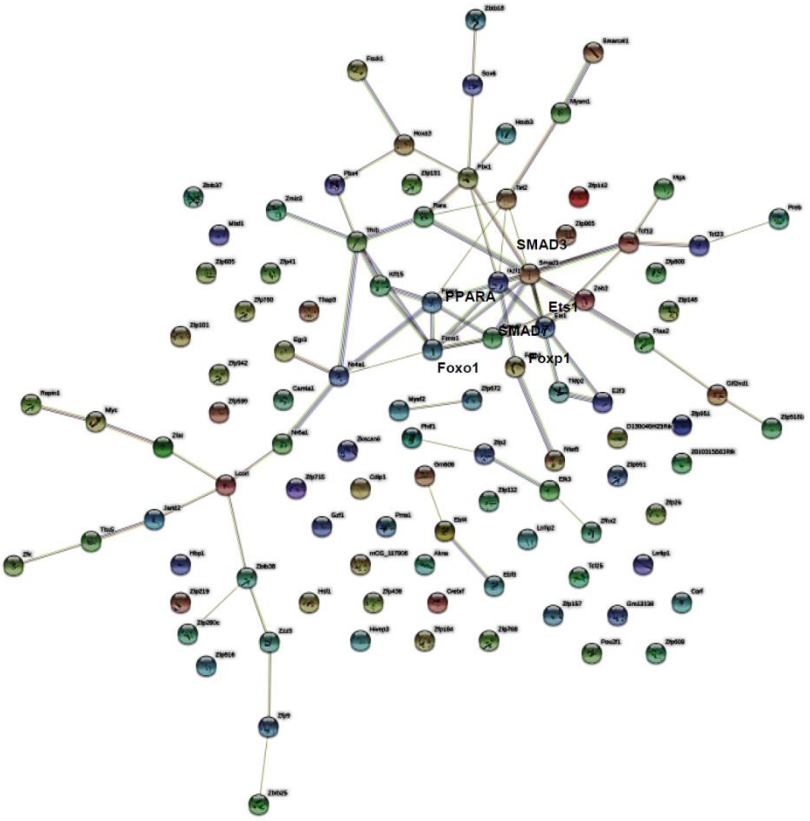

Supplementary Fig. 9: Foxo$^{fl/fl}$.CD4^{Cre+} mice shows protective immune response by distinct transcriptional signatures associated with SARS-CoV-2 infection

a-b) mRNA expression of *Il9, Il4, Il5, Il13, Gata3, Batf* and *Pu.1* was determined by qPCR from infected lung samples, *P<0.05,**P<0.005, ***p<0.0005 ****P<0.0001(One-way ANOVA-Tukey's multiple comparison test (n=4); Bar graph represents as a mean $\pm$SEM. **c**) Percent frequency of IL-17^{+} and IFN-γ^{+} cells gated on CD4^{+} T cells by flow cytometry. n=5 mice; Bar graph represents as a mean $\pm$SEM; one-way ANOVA followed by Tukey's multiple comparison test**.** Total RNA was isolated from UI, ACE2.Tg infected, and Foxo$^{fl/fl}$.CD4^{Cre+} infected mice for transcriptomic analysis. **d)** PCA analysis. **e)** Volcano plots comparing differentially expressed genes between I Vs UI and Foxo$^{fl/fl}$.CD4^{Cre+} _I Vs ACE2.Tg_I. Red and green indicate upregulated and downregulated genes, respectively, with a fold change > 2 and a false discovery rate < 0.05. **f)** Venn diagram showing overlap between genes up and down regulated in the hACE2.Tg mice and Foxofl/fl.CD4Cre+ infected mice. **g)** Pathway enrichment analysis of genes up and down-regulated from comparisons of hACE2.Tg versus Foxo1$^{fl/fl}$CD4^{Cre+} infected mice. **h)** Heat map of all significantly differentially expressed top 100 genes (DEGs) between uninfected and infected mice; and further heat-map analysis of selected top significantly differentially expressed genes in asthmatic and lung injury. **i, j)** String analysis (Version 10.5) showing the key transcriptional regulators that are activated, (e) and inhibited in Foxo$^{fl/fl}$.CD4^{Cre+} infected mice as compared to ACE2.Tg infected mice.

Supplementary Fig. 10

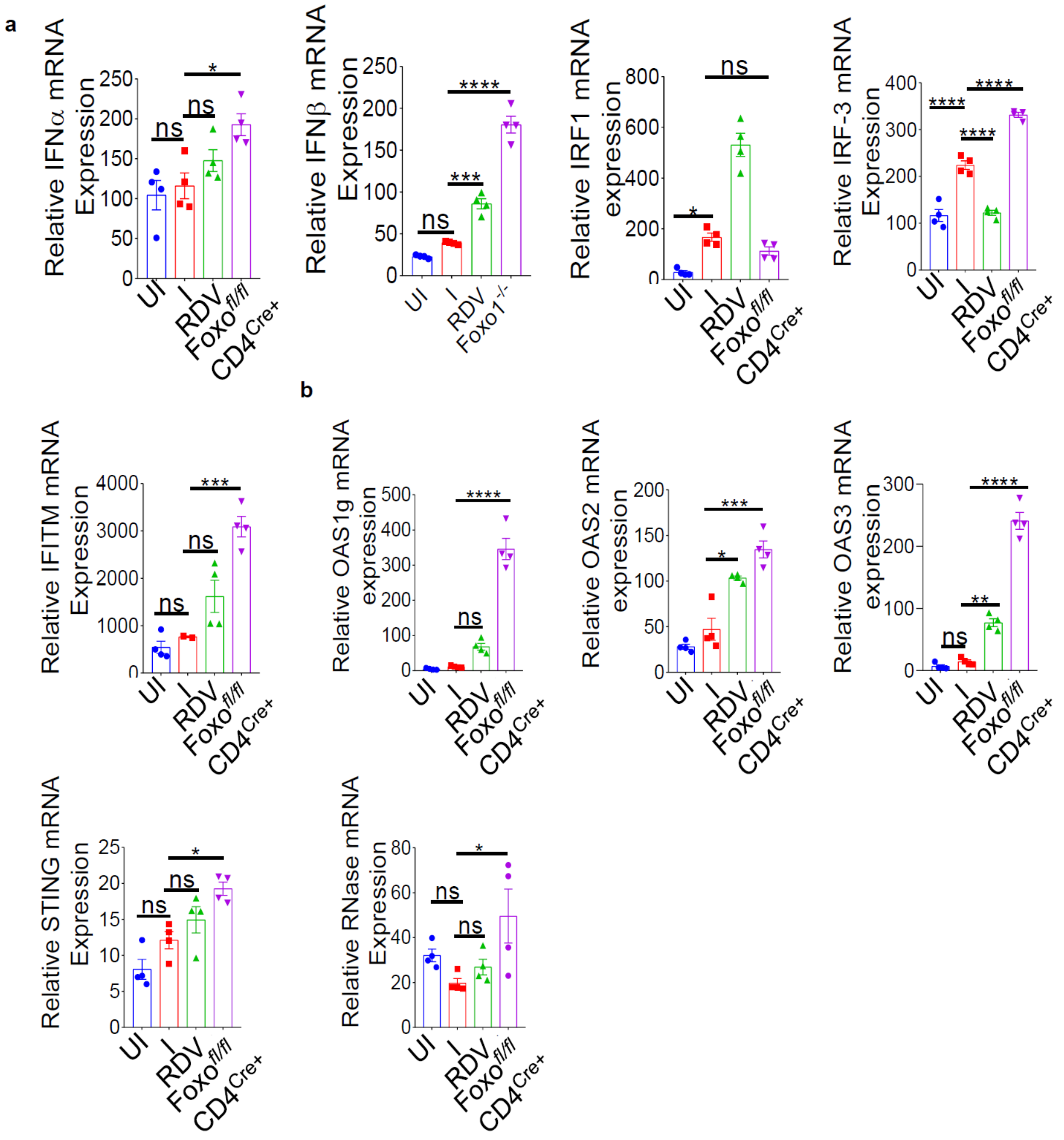

Supplementary Fig. 10: Foxofl/fl.CD4Cre+ Mice shows defensive anti-viral response and ISGs.

a-b) Relative mRNA expression of ISGs genes (*Ifn-α, Ifn-β, Irf-1, Irf-3, Ifitm, Sting*) and Anti-viral genes (*Oas1g, Oas2, Oas3 and RNaseL*) was measured by RT-PCR; bar graph represents as a mean ±SEM (n=4 mice per group); one-way ANOVA followed by Tukey's multiple comparison test (*p<0.05, **p<0.005, ***p<0.002, ****p<0.0001).

a

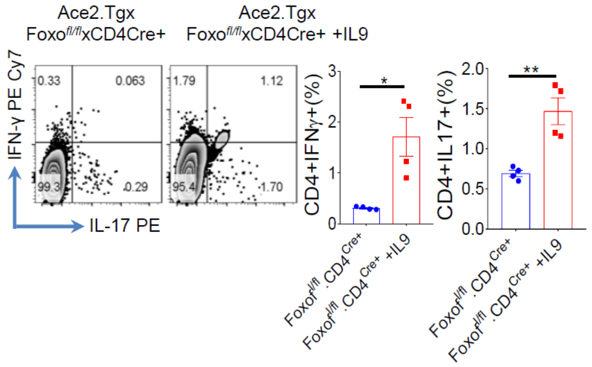

Supplementary Fig. 11: rIL9 makes Foxo$^{fl/fl}$.CD4^{Cre+} mice susceptible to SARS-CoV-2 infection

a) Representative FACS plot and its corresponding bar graph showing percent frequency of IFN-γ and IL-17 in BAL; bar graph represents as a mean ±SEM; (n=4 mice per group); (*p<0.05, **p<0.005; students t test).

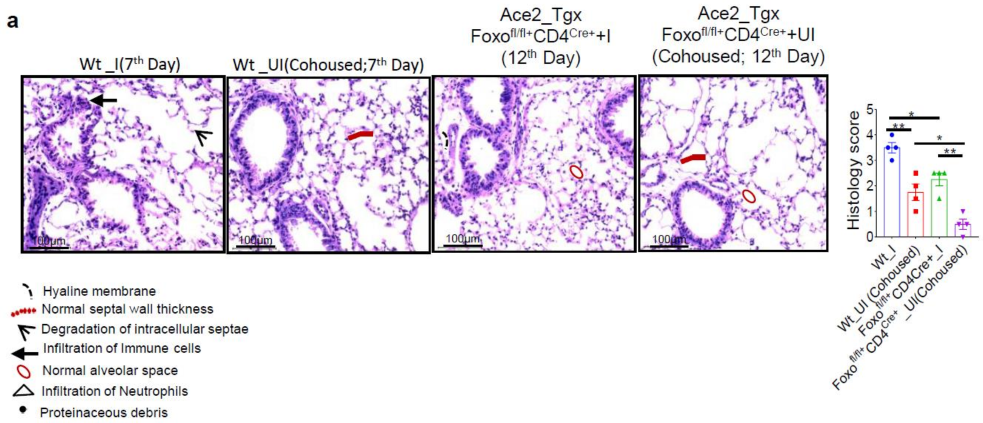

Supplementary Fig. 12: Histological analysis of cohoused mice

a) HE staining (100µm) shows that, Foxofl/fl.CD4Cre+ infected mice and hACE2.Tg_UI mice showed significantly lower histopathological scores compared to wt mice. Bar graph represents $\pm$ SEM; one-way ANOVE followed by Tukey's test (n=4 mice per group); experiment was performed once; *p<0.05, **p<0.005.

Supplementary Fig. 13

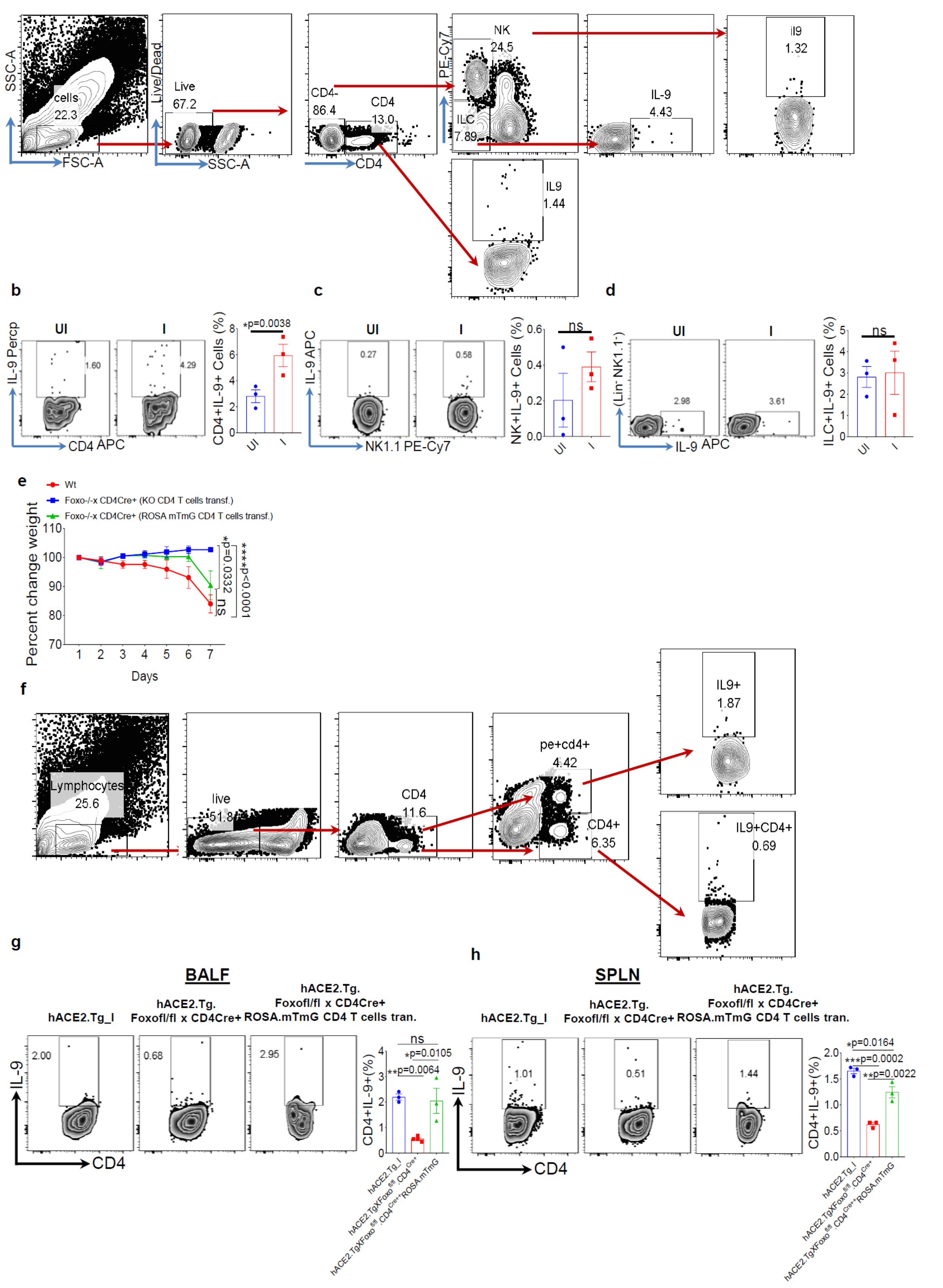

Supplementary Fig. 13: Adoptive transfer of wt CD4⁺ T cells makes hACE2 Tg.Foxo$^{fl/fl}$.CD4^{Cre+} mice susceptible to infection

a) Gating strategy to identify IL-9-producing ILCs, NK and CD4+ T cells. **b-d)** Zebra plots shows the percentage of IL-9 secreting CD4, NK and ILCS; n=3 mice per group; (Error bar represents $\pm$ SEM; Students t test) *p=0.0038, ns=non-significant. To determine the role of $CD4^+$ T cells, we sorted the ROSA mTmG mice $CD4^+$ T cells (>95% purity) and $Foxo^{fl/fl}$x $CD4^{Cre+}$ mice CD4 T cells (>95% purity) by FACS Aria III. For adoptive transfer, cells were suspended in PBS. $CD4^+$ T cells (0.5 x 10^6) from ROSA mTmG or $Foxo^{fl/fl}$x$CD4^{Cre+}$ mice were injected intraperitoneally (200µl) into the $Foxo^{fl/fl}$x$CD4^{Cre+}$ mice in individual groups (n=4 mice per group). After 6 days, both groups were infected with 10^3 PFU of SARS-CoV2 ancestral strain. We followed up for the percent body weight change of these mice post challenge. We found that mice that received ROSA mTmG CD4+ T cells lost significant body weight compared to those that received $Foxo^{fl/fl}$x $CD4^{Cre+}$ $CD4^+$ T cells (two-way ANOVA; bar represents as a mean $\pm$ SEM) **(e)**. Gating strategy for the ROSA mTmG $CD4^+$ T cells **(f)**; we further characterized the percentage of IL-9 in these groups. We found that the percentage of IL-9 were significantly increased in mTmG CD4 adaptive T cell transferred group compared to $Foxo^{fl/fl}$x$CD4^{Cre+}$ T cells transferred group (n=3 mice per group; one-way ANOVA followed by Tukey's multiple comparison test); Bar graph represents as a mean $\pm$ SEM; *p<0.05, **p<0.005, ***p<0.0005, ns= non-significant **(g-h)**.

Supplementary Table. 1: Description of the COVID-19 and Healthy individuals

		COVID-19 Positive	Healthy Control
Number of Donors		9	9
Gender	Male	5	5
	Female	4	4
Age (Median and range)		35 (30:42)	29 (25:35)
Symptoms	Maligia	5	
	Fever	9	
	Dry Cough	6	
	ARDS	4	
	Gastric Upset	2	
Days post PCR positive report		0-3 days	
In-hospital or Day-28-mortality		1	

Supplementary Table.1. Description of the COVID-19 positive and Healthy individual subjects. Blood samples were collected as per the recommended guidelines of the Institutional Ethics Committee of THSTI (Human Research) and ESIC Hospital, Faridabad (Letter Ref No: THS 1.8.1/ (97) dated July 07, 2020).

Supplementary Table 2: Minor variant analysis of ancestral SARS-CoV-2 shows the SNVs present in the ancestral SARS-CoV-2 and Omicron strain.

SARS-CoV-2

Details of observed point mutations in the sample												
Sample	Chr	Position	RefBase	Gene	Coverage	AvQual	A	T	G	C	strandbias	maf
SARS-CoV-2 (GenBank: MN908947.3)	NC_045512.2	22205	G	spike_glycoprotein	661	0.070829	9	6	646	0	A7:2;C0:0;T6:0;G473:173	0.12361457
SARS-CoV-2 (GenBank: MN908947.3)	NC_045512.2	22295	C	spike_glycoprotein	598	0.066742	0	6	1	591	A0:0;C493:98;T4:2;G0:1	0.068500749
SARS-CoV-2 (GenBank: MN908947.3)	NC_045512.2	27945	C	ORF8	445	0.076611	0	245	1	199	A0:0;C5:194;T15:230;G0:1	0.702173335
SARS-CoV-2 (GenBank: MN908947.3)	NC_045512.2	27999	C	ORF8	456	0.068356	0	6	1	449	A0:0;C41:408;T0:6;G0:1	0.085642298
SARS-CoV-2 (GenBank: MN908947.3)	NC_045512.2	28143	T	ORF8	937	0.039352	2	915	4	16	A2:0;C1:15;T562:353;G4:0	0.12912041

Omicron (B.1.1.529)

Details of observed point mutations in the sample												
Sample	Chr	Position	RefBase	Gene	Coverage	AvQual	A	T	G	C	strandbias	maf
Omicron (B.1.1.529)	NC_045512.2	241	C	five_prime_UTR	240	0.000317	0	215	0	25	A0:0;C15:10;T106:109;G0:0	0.104166667
Omicron (B.1.1.529)	NC_045512.2	2832	A	ORF1ab	1029	0.000813	119	0	910	0	A70:49;C0:0;T0:0;G448:462	0.115646259
Omicron (B.1.1.529)	NC_045512.2	9358	T	ORF1ab	2246	0.000876	0	2227	0	19	A0:0;C8:11;T1133:1094;G0:0	0.008459484
Omicron (B.1.1.529)	NC_045512.2	10449	C	ORF1ab	1580	0.00097	1412	0	0	168	A699:713;C75:93;T0:0;G0:0	0.106329114
Omicron (B.1.1.529)	NC_045512.2	11124	C	ORF1ab	1848	0.00114	3	18	0	1827	A2:1;C907:920;T10:8;G0:0	0.009756098
Omicron (B.1.1.529)	NC_045512.2	11537	A	ORF1ab	2444	0.000749	91	2	2351	0	A48:43;C0:0;T0:2;G1167:1184	0.037264537
Omicron (B.1.1.529)	NC_045512.2	13841	T	ORF1ab	1800	0.000663	0	1783	17	0	A0:0;C0:0;T900:883;G15:2	0.009444444
Omicron (B.1.1.529)	NC_045512.2	15060	T	ORF1ab	785	0.001316	0	749	0	36	A0:0;C17:19;T391:358;G0:0	0.045859873
Omicron (B.1.1.529)	NC_045512.2	15240	C	ORF1ab	1044	0.000863	3	728	0	313	A2:1;C169:144;T377:351;G0:0	0.30067243
Omicron (B.1.1.529)	NC_045512.2	15652	G	ORF1ab	1423	0.001052	15	2	1406	0	A10:5;C0:0;T0:2;G692:714	0.010555947
Omicron (B.1.1.529)	NC_045512.2	18747	C	ORF1ab	913	0.000764	0	21	0	892	A0:0;C454:438;T10:11;G0:0	0.023001095
Omicron (B.1.1.529)	NC_045512.2	20956	C	ORF1ab	979	0.000851	0	205	0	774	A0:0;C417:357;T103:102;G0:0	0.209397344
Omicron (B.1.1.529)	NC_045512.2	21077	C	ORF1ab	1124	0.000592	0	148	0	976	A0:0;C471:505;T69:79;G0:0	0.131672598
Omicron (B.1.1.529)	NC_045512.2	22813	G	spike_glycoprotein	201	0.001481	0	173	28	0	A0:0;C0:0;T102:71;G10:18	0.139303483
Omicron (B.1.1.529)	NC_045512.2	22882	T	spike_glycoprotein	341	0.001156	0	51	290	0	A0:0;C0:0;T31:20;G136:154	0.149560117
Omicron (B.1.1.529)	NC_045512.2	22898	G	spike_glycoprotein	289	0.001129	266	0	23	0	A135:131;C0:0;T0:0;G17:6	0.079584775
Omicron (B.1.1.529)	NC_045512.2	23202	C	spike_glycoprotein	345	0.000888	263	0	0	164	A131:132;C35:47;T0:0;G0:0	0.137681159
Omicron (B.1.1.529)	NC_045512.2	26530	A	M_gene	2150	0.001096	1139	3	1007	1	A529:610;C0:1;T1:2;G486:521	0.469245107
Omicron (B.1.1.529)	NC_045512.2	26577	C	M_gene	3231	0.000571	1	1	2530	699	A0:1;C363:336;T1:0;G1299:1231	0.216475689
Omicron (B.1.1.529)	NC_045512.2	26951	G	M_gene	4737	0.001573	1	18	4716	2	A0:1;C1:1;T7:11;G2426:2290	0.003802281
Omicron (B.1.1.529)	NC_045512.2	27925	C	ORF8	1191	0.000679	2	22	0	1167	A1:1;C572:595;T9:13;G0:0	0.018502944
Omicron (B.1.1.529)	NC_045512.2	28853	T	N_gene	2224	0.001495	37	2185	0	2	A17:20;C0:2;T1129:1056;G0:0	0.016651665
Omicron (B.1.1.529)	NC_045512.2	28854	C	N_gene	2112	0.000893	27	0	0	2085	A16:11;C1063:1022;T0:0;G0:0	0.012784091

**Sample = Name of the SARSCoV2 variant**
**Chr = Reference genome**
**Position = Position of nucleotide in the reference genome**
**RefBase = Nucleotide base in the reference genome**
**Gene = Name of the reference's gene**
**Coverage = Number of aligned reads at the position**
**AvQual = Average read's quality**
**A = Number of adenine base**
**T = Number of thymine base**
**G = Number of guanine base**
**C = Number of cytosine base**
**strandbias = number of each base covered by either forword or reverse reads (separated by :)**
**Maf = Minor allele frequency**

Supplementary Table.2. SARSCoV2 and Omicron (B.1.1.529) raw reads were mapped to Wuhan's SARS-CoV2 and Omicron sequence to generate a consensus genome. A BAM file was generated and processed to find highly accurate SNVs with read depth ≥15 and phred score ≥30. We found 5, 24 SNVs in SARS-CoV-2 and Omicron respectively.

Supplementary Movie 1:

Overall activity of SARS-CoV-2 infected Foxo1$^{fl/fl}$. CD4^{Cre+} mice. A short video clip was made to record the overall activity of SARS-CoV-2 infected Foxo1$^{fl/fl}$. CD4^{Cre+} mice. It is clearly shown that Foxo1$^{fl/fl}$. CD4^{Cre+} mice were remained active post SARS-CoV-2 infection

Supplementary Movie 2:

Overall activity of SARS-CoV-2 infected x Foxo1$^{fl/fl}$ .CD4^{Cre-} mice

A short video clip was made to record the overall activity of SARS-CoV-2 infected x Foxo1$^{fl/fl}$ .CD4^{Cre-} mice. It is clearly shown that Foxo1$^{fl/fl}$. CD4^{Cre-} mice were clearly shows SARS-CoV-2 infection as compared to Foxo1$^{fl/fl}$. CD4^{Cre+} mice.

Supplementary Movie 3: Overall activity of SARS-CoV-2 infected Foxo1$^{fl/fl}$. CD4^{Cre+} mice in absence of exogenous IL-9 treatment mice.

A short video clip was made to record the overall activity of SARS-CoV-2 infected Foxo1$^{fl/fl}$. CD4^{Cre+} treated with exogenous IL-9 intranasally. It is clearly shown that Foxo1$^{fl/fl}$. CD4^{Cre+} mice were remained active as compared to Foxo1$^{fl/fl}$. CD4^{Cre+} mice treated with exogenous IL-9 post SARS-CoV-2 infection.

Supplementary Movie 4: Overall activity of SARS-CoV-2 infected Foxo1$^{fl/fl}$. CD4^{Cre+} treated with exogenous IL-9 treatment mice. A short video clip was made to record the overall activity of SARS-CoV-2 infected Foxo1$^{fl/fl}$. CD4^{Cre+} treated with exogenous IL-9 intranasally.
