# Supplementary material for: IL-9 aggravates SARS-CoV-2 infection and exacerbates associated airway inflammation
Source: Nat Commun. 2023 Jul 10;14:4060. doi: 10.1038/s41467-023-39815-5 (PMC10333319; doi:10.1038/s41467-023-39815-5)
Supplement: Supplementary file 3 — Description of Additional Supplementary Files [file 41467_2023_39815_MOESM3_ESM.pdf]

Description of Additional Supplementary Files

File Name: Supplementary Movie 1
Description: **Overall activity of SARS-CoV-2 infected Foxo1$^{fl/fl}$. CD4^{Cre+} mice.** A short video clip was made to record the overall activity of SARS-CoV-2 infected Foxo1$^{fl/fl}$. CD4^{Cre+} mice. It is clearly shown that Foxo1$^{fl/fl}$. CD4^{Cre+} mice were remained active post SARS-CoV-2 infection

File Name: Supplementary Movie 2
Description: **Overall activity of SARS-CoV-2 infected x Foxo1$^{fl/fl}$ .CD4^{Cre-} mice.** A short video clip was made to record the overall activity of SARS-CoV-2 infected x Foxo1$^{fl/fl}$ .CD4^{Cre-} mice. It is clearly shown that Foxo1$^{fl/fl}$. CD4^{Cre-} mice were clearly shows SARS-CoV-2 infection as compared to Foxo1$^{fl/fl}$. CD4^{Cre+} mice.

File Name: Supplementary Movie 3
Description: **Overall activity of SARS-CoV-2 infected Foxo1$^{fl/fl}$. CD4^{Cre+} mice in absence of exogenous IL-9 treatment mice.** A short video clip was made to record the overall activity of SARS-CoV-2 infected Foxo1$^{fl/fl}$. CD4^{Cre+} treated with exogenous IL-9 intranasally. It is clearly shown that Foxo1$^{fl/fl}$. CD4^{Cre+} mice were remained active as compared to Foxo1$^{fl/fl}$. CD4^{Cre+} mice treated with exogenous IL-9 post SARS-CoV-2 infection.

File Name: Supplementary Movie 4
Description: **Overall activity of SARS-CoV-2 infected Foxo1$^{fl/fl}$. CD4^{Cre+} treated with exogenous IL-9 treatment mice.** A short video clip was made to record the overall activity of SARS-CoV-2 infected Foxo1$^{fl/fl}$. CD4^{Cre+} treated with exogenous IL-9 intranasally.
