# Supplementary material for: IL-9 aggravates SARS-CoV-2 infection and exacerbates associated airway inflammation
Source: Nat Commun. 2023 Jul 10;14:4060. doi: 10.1038/s41467-023-39815-5 (PMC10333319; doi:10.1038/s41467-023-39815-5)
Supplement: Supplementary file 8 — Reporting Summary [file 41467_2023_39815_MOESM8_ESM.pdf]

nature portfolio

Corresponding author(s): Dr. Amit Awasthi

Last updated by author(s): Apr 22, 2023

Reporting Summary

Nature Portfolio wishes to improve the reproducibility of the work that we publish. This form provides structure for consistency and transparency in reporting. For further information on Nature Portfolio policies, see our Editorial Policies and the Editorial Policy Checklist.

Statistics

For all statistical analyses, confirm that the following items are present in the figure legend, table legend, main text, or Methods section.

n/a	Confirmed	
☐	☒	The exact sample size (*n*) for each experimental group/condition, given as a discrete number and unit of measurement
☐	☒	A statement on whether measurements were taken from distinct samples or whether the same sample was measured repeatedly
☐	☒	The statistical test(s) used AND whether they are one- or two-sided<br>*Only common tests should be described solely by name; describe more complex techniques in the Methods section.*
☐	☒	A description of all covariates tested
☐	☒	A description of any assumptions or corrections, such as tests of normality and adjustment for multiple comparisons
☐	☒	A full description of the statistical parameters including central tendency (e.g. means) or other basic estimates (e.g. regression coefficient) AND variation (e.g. standard deviation) or associated estimates of uncertainty (e.g. confidence intervals)
☐	☒	For null hypothesis testing, the test statistic (e.g. *F*, *t*, *r*) with confidence intervals, effect sizes, degrees of freedom and *P* value noted<br>*Give P values as exact values whenever suitable.*
☒	☐	For Bayesian analysis, information on the choice of priors and Markov chain Monte Carlo settings
☒	☐	For hierarchical and complex designs, identification of the appropriate level for tests and full reporting of outcomes
☒	☐	Estimates of effect sizes (e.g. Cohen's *d*, Pearson's *r*), indicating how they were calculated

Our web collection on statistics for biologists contains articles on many of the points above.

Software and code

Policy information about availability of computer code

Data collection

1. Flow cytometry data were collected on BD FACSCanto II with FACSDiva software version 8.0.2 (BD).
2. qRT-PCR data were collected on Fast 7500 Dx qPCR system (Applied Biosystems) and analyzed using SDS 2.1 software.
3. ELISA data were collected at 600nm in spectrophotometer (BioLinkk).
4. RNA sequencing of SARS-CoV-2 infected lungs of ACE2.Tg, hACE2.Tgx Foxofl/fl.CD4Cre-, and Foxofl/fl.CD4Cre+ SARS-CoV-2 infected lung tissues were homogenised and RNA was derived and subjected to next-generation sequencing (NGS) to generate deep coverage RNASeq data. Size selection of RNA fragments was done with SPRI Beads-based Size Selection. High quality libraries were prepared using NEB Next Ultra II Directional RNA Library Prep Kit according to manufacturer's protocols and paired-end reads of 151bp read length were generated on the Illumina Novoseq 6000 platform. Transcriptome analysis is mentioned in methodology section in detail.

Data analysis

1- Flow cytometry data were analyzed on FlowJo version 10(BD).
2. All qPCR data was analyzed using SDS 2.1 software and microsoft excel.
3. Quality based filtering and adapter trimming of the raw sequencing reads was done using fastp (v0.20.1). A thresh hold of 30 was set for the phred quality score. The filtered reads were aligned against the Mus musculus (mm39) genome using the splice aware aligner Hisat (v2.2.1).
The alignments were assembled into transcripts with stringtie assembler (v2.1.5). Stringtie computes read counts for the genes and normalized expression values with the Transcript per million (TPM) metric. The gene read counts were used for differential analysis between the conditions. Genes having a p-value of less than 0.05 were considered to have a significant differential change in the expression between the conditions. A log2Foldchange of 2 and higher of these significant genes were classified as up-regulated and a log2Foldchange of -2 and lower as down-regulated. Genes were functionally annotated with Gene Ontology terms and Reactome pathways using NCBI resources. David Bioinformatics resources (v6.8) was used to identify significant enrichment of significant GO terms and pathways. String database was used to

determine for interaction of the protein-coding genes. A high confidence score of 0.9 was used to compute the interactions. R packages used for visualization - Complex Heat map, Enhanced Volcano, ggplot2.
4. All the statistical analysis were performed on Prism version 8 (GraphPad).
5. MS Office 2016 was used for making excel file, word file and ppt files.
6. The primer-free pair raw reads of SARSCoV2 were generated from the Illumina MiSeq. Raw reads of SARSCoV2 were pre-processed based on read quality and read length (phred quality ≥30 and minimum length ≥ 50 base pair) and merged by PEAR program. The merged reads were mapped to Wuhan's SARSCoV2 sequence (Genebank ID: NC_045512.2) to generate a consensus genome. During the mapping of reads to reference genome sequence, a BAM file was generated by Samtools. This BAM file was processed by diversiutils script in DiversiTools (http://josephhughes.github.io/btctools/) to find the frequency of all types of four bases for each position of a reference sequence. The only variants that have been covered by at least 15 times (read depth ≥ 15) by high-quality reads (average read's phred score ≥ 30) to find highly accurate single nucleotide variants (SNVs)

For manuscripts utilizing custom algorithms or software that are central to the research but not yet described in published literature, software must be made available to editors and reviewers. We strongly encourage code deposition in a community repository (e.g. GitHub). See the Nature Portfolio guidelines for submitting code & software for further information.

Data

Policy information about availability of data

All manuscripts must include a data availability statement. This statement should provide the following information, where applicable:

- Accession codes, unique identifiers, or web links for publicly available datasets
- A description of any restrictions on data availability
- For clinical datasets or third party data, please ensure that the statement adheres to our policy

Data Availability Statement: All Data needed to evaluate the conclusions in the paper are present in the paper and/or the Supplementary Materials. The RNA sequence Data generated in this study has been deposited in the NCBI SRA database under the accession code no. PRJNA842504. Publicly available data with accession code, GSE209550 (https://www.ncbi.nlm.nih.gov/geo/query/acc.cgi?acc=GSE209550). The authors declare that, the necessary data required to validate the findings of the paper can be found within the article itself or in the Supplementary Materials. Source data are provided with this paper.
The authors declare that all other data supporting the findings of this study are available within the article and its supplementary information files.

Research involving human participants, their data, or biological material

Policy information about studies with human participants or human data. See also policy information about sex, gender (identity/presentation), and sexual orientation and race, ethnicity and racism.

Reporting on sex and gender	COVID-19 patient blood samples were collected from 4 female (Median age = 33.5y) and 5 males (median age = 35y). For comparison blood from healthy COVID-19 negative volunteers were collected after consent forms were signed (Females (n= 4; median age = 33y; Males: n=5,median age = 29y). Finding does not apply for a specific Sex or Gender.
Reporting on race, ethnicity, or other socially relevant groupings	NA
Population characteristics	The COVID-19 patient cohort (n=9) was a median of 26.5 years old (1OR 24:34.5). Blood samples were collected within 0-4 days of PCR positivity. Similarly, blood was collected from age-matched RT-PCR negative, healthy individuals [n=9; median age 27.5 years (IQR 25:32y)].
Recruitment	Participants were enrolled according to the inclusion/exclusion criteria set by IAEC of THSTI and ESIC hospital. Inclusion/Exclusion criteria: The study recruited individuals between the ages of 18 to 60 years who were symptomatic and had RT-PCR-confirmed COVID-19. Additionally, age-matched healthy individuals who tested negative for RT-PCR were also recruited. Written informed consent was obtained from all participants, and 10 ml of blood was collected by phlebotomy in sodium heparin tubes and stored briefly at room temperature before processing for PBMC and plasma isolation. Immunocompromised individuals were excluded from the study as their immune response may be affected. Samples were obtained within 0 to 3 days of a positive PCR test. The recruitment and collection process was unbiased.
Ethics oversight	Active COVID-19 patients' blood samples were collected during the first wave of SARS-CoV-2 in mid-2020 after obtaining a approval from the Institutional Ethics Committee of THSTI (IEC, Human Research) and ESIC Hospital, Faridabad (Letter Ref No: THS 1.8.1/ (97) dated July 07, 2020). Human peripheral blood samples were collected from symptomatic COVID-19 patients and healthy participants after the written informed consent and there was no bias to the recruitment or collection. PBMCs were isolated from collected blood samples, and stored in liquid nitrogen as mentioned in methodology section.<br>The study was approved by the Institutional Ethics Committee (Human Research) of THSTI and ESIC Hospital, Faridabad (Letter Ref No: THS 1.8.1/ (97) dated 07th July 2020). Human peripheral blood samples were collected from symptomatic COVID-19 patients and healthy participants after the written informed consent.

Note that full information on the approval of the study protocol must also be provided in the manuscript.

Field-specific reporting

Please select the one below that is the best fit for your research. If you are not sure, read the appropriate sections before making your selection.

☒ Life sciences ☐ Behavioural & social sciences ☐ Ecological, evolutionary & environmental sciences

For a reference copy of the document with all sections, see nature.com/documents/nr-reporting-summary-flat.pdf

Life sciences study design

All studies must disclose on these points even when the disclosure is negative.

Sample size	The sample size for in vivo studies were determined based on our preliminary data. The sample size was selected to produce statistically relevant biological difference in the study. Sample sizes were determined in accordance with the literature and based on previous experience in our group.
Data exclusions	No data were excluded from the analyses.
Replication	All the experiments were replicated at least 3 times independently. For all in vivo studies biological replicates were taken.
Randomization	Randomizations of the mice were done based on their body weight or genotypes. mice of different genotypes were randomly assigned to treatment groups throughout the study. For experiments involving genetically modified animals, litter mates were used for each experiment.
Blinding	To evaluate unbiased disease phenotype blinding was done by 3 unbiased observers. Histological score was given by professional histologist for n=5 samples. Other data presented did not require the use of blinding. Data reported for mouse experiments were not subjective but rather based on quantitative analyses. In the cell and animal experiments, investigators were not blinded to group allocation because the investigators should give the drug to the mice and cell in different treatment conditions. For flowcytometry experiments, the samples were acquired blindly.

Reporting for specific materials, systems and methods

We require information from authors about some types of materials, experimental systems and methods used in many studies. Here, indicate whether each material, system or method listed is relevant to your study. If you are not sure if a list item applies to your research, read the appropriate section before selecting a response.

Materials & experimental systems

n/a	Involved in the study	
☐	☒	Antibodies
☐	☒	Eukaryotic cell lines
☒	☐	Palaeontology and archaeology
☐	☒	Animals and other organisms
☒	☐	Clinical data
☒	☐	Dual use research of concern
☒	☐	Plants

Methods

n/a	Involved in the study	
☒	☐	ChIP-seq
☐	☒	Flow cytometry
☒	☐	MRI-based neuroimaging

Antibodies

Antibodies used	The following antibodies were used: anti-mouse CD3 BV510 (Cat no- 100353,Clone-145-2C11,Biolegend INC, USA, 2:1000), anti-mouse γδTCR FITC (Cat no-118106,Clone-GL3, , Biolegend INC,USA, 2:1000), anti-mouse Gr1 BV421 (Cat no-108445,Clone-RB6-8C5, Biolegend INC, USA, 2:1000), anti-mouse CD11b PerCp-Cy5.5 (Cat no-101228, Biolegend INC, USA, 1:1000), anti-mouse CD4-Percp cy5.5 (Cat no-100538,Clone-RM4-5, Biolegend INC, USA, 1:1000), anti-mouse CD4-FITC (#100406; Clone-GK1.5, Biolegend, INC, USA, 1:1000), anti-mouse NK1.1–PE-Cy7 (Cat no-108714,Clone-PK136, Biolegend INC, USA, 1:1000), anti-mouse-CD8 – BV421 (Cat no-100753,Clone-53-6.7 Biolegend INC, USA,2:2000), F4/80 – FITC (Cat no-123108,Clone-BM8,Biolegend INC, USA, 1:1000), CD206 - PE(Cat no-141705,Clone-C068C2, Biolegend INC, USA,2:2000), CD80 – AF647 (Cat no-305216,Clone-2D10, Biolegend INC, USA, 2:2000), CD68 – PEcy7(#137015,Clone-FA-11, Biolegend INC, USA, 1:1000), CD49b (#117322,Clone-N418, Biolegend INC, USA, 1:1000), C-kit (#105805,Clone-2B8, Biolegend INC, USA, 1:1000), Fcer1 (#134308, Clone-MAR1, Biolegend INC, USA, 1:1000),Siglec-f(#155528,Clone-S17007L, Biolgend INC, USA, 1:1000), IFNγ – AF647 (#505814,Clone-XMG1.2, Biolegend INC, USA, 1:1000), IL-17 – PE-cy7 (#506922, Clone- TC11-18H10.1,Biolegend INC, USA, 1:1000), IL-10 – PE (#505008, Clone- JES5-16E3, Biolegend INC, USA, 5:1000), Foxp3 – AF647 (#126408,Clone-MF14, Biolegend, USA,2:1000), IL-9 – Percp-cy5.5 (#514112,Clone-RM9A4, Biolegend INC, USA, 5:1000), IL-4 – PE(#504104, Clone-11B11,Biolegend, USA, 1:1000), anti-mouse CD3 FITC (# 100204, Clone-17A2, Biolegend INC, USA, 2:2000), anti-mouse CD11b FITC (#101206,Clone-M1/70, Biolegend INC,USA, 1:1000), F4/80 – FITC (#123108, Clone-BM8, Biolegend INC, USA, 1:1000), anti-mouse B220- FITC (#103206,Clone-RA3-6B2, Biolegend INC, USA, 2:2000), anti-mouse CD4-Percp cy5.5 (100538,Clone-RM4-5, Biolegend INC, USA, 1:1000), anti-mouse NK1.1–PE-Cy7 (108714,Clone-PK136, Biolegend INC, USA, 1:1000), IL-9 – APC (#514106, Clone-RM9A4, Biolegend, USA, 3:3000), IL-4 – PE-Cy7 (#504118, Clone-11B11, Biolegend, USA, 1:200)

Validation

All antibodies were validated by the supplier (Bio Legend, Peprotech) and were checked in the lab by comparing manufacturers or in house results. Statement from Bio legend: Bio legend Antibodies under go an extensive series of testing to ensure quality at every step in the manufacturing process, as well as maintaining quality after the sale.
Statement from Peprotech: Peprotechs monoclonal antibodies are raised against full length recombinant antigens and have been thoroughly screened for performance in variety of applications.
The validation statement and the relevant citation information is listed in the link:
1. anti-mouse CD3 BV510 (Cat no- 100353,Clone-145-2C11,Biolegend INC, USA, 2:1000) https://www.biolegend.com/en-ie/products/brilliant-violet-510-anti-mouse-cd3epsilon-antibody-11973
2. anti-mouse γδTCR FITC (Cat no-118106,Clone-GL3, , Biolegend INC,USA, 2:1000) https://www.biolegend.com/en-us/products/fitc-anti-mouse-tcr-gamma-delta-antibody-2420?GroupID=BLG3687
3. anti-mouse Gr1 BV421 (Cat no-108445,Clone-RB6-8C5, Biolegend INC, USA, 2:1000) https://www.biolegend.com/en-us/products/brilliant-violet-421-anti-mouse-ly-6g-ly-6c-gr-1-antibody-7201?GroupID=BLG4876
4. anti-mouse CD11b PerCp-Cy5.5 (Cat no-101228, Biolegend INC, USA, 1:1000) https://www.biolegend.com/en-us/products/percp-cyanine5-5-anti-mouse-human-cd11b-antibody-4257?GroupID=BLG10552
5. anti-mouse CD4-Percp cy5.5 (Cat no-100538,Clone-RM4-5, Biolegend INC, USA, 1:1000) https://www.biolegend.com/en-us/products/percp-cyanine5-5-anti-mouse-cd4-antibody-4230?GroupID=BLG4211
6. anti-mouse NK1.1–PE-Cy7 (Cat no-108714,Clone-PK136, Biolegend INC, USA, 1:1000) https://www.biolegend.com/en-us/products/pe-cyanine7-anti-mouse-nk-1-1-antibody-2840?GroupID=GROUP20
7. anti-mouse-CD8 – BV421 (Cat no-100753,Clone-53-6.7 Biolegend INC, USA,2:2000) https://www.biolegend.com/en-us/products/brilliant-violet-421-anti-mouse-cd8a-antibody-7138?GroupID=BLG6765
8. F4/80 – FITC (Cat no-123108,Clone-BM8,Biolegend INC, USA, 1:1000) https://www.biolegend.com/en-us/products/fitc-anti-mouse-f4-80-antibody-4067?GroupID=BLG5319
9. CD206 - PE(Cat no-141705,Clone-C068C2, Biolegend INC, USA,2:2000) https://www.biolegend.com/en-us/products/pe-anti-mouse-cd206-mmr-antibody-7424?GroupID=BLG9506
10. CD80 – AF647 (Cat no-305216,Clone-2D10, Biolegend INC, USA, 2:2000) https://www.biolegend.com/en-us/products/alexa-fluor-647-anti-human-cd80-antibody-3352?GroupID=BLG1908
11. CD68 – PEcy7 (#137015,Clone-FA-11, Biolegend INC, USA, 1:1000) https://www.biolegend.com/en-us/products/pe-cyanine7-anti-mouse-cd68-antibody-9124?GroupID=BLG10716
12. CD49b (#117322,Clone-N418, Biolegend INC, USA, 1:1000) https://www.biolegend.com/en-us/products/pe-anti-mouse-cd49b-antibody-299?GroupID=BLG4895
13. C-kit (#105805,Clone-2B8, Biolegend INC, USA, 1:1000) https://www.biolegend.com/en-us/products/purified-anti-mouse-cd117-c-kit-antibody-77?GroupID=BLG4276
14. Fcer1 (#134308, Clone-MAR1, Biolegend INC, USA, 1:1000) https://www.biolegend.com/en-us/products/pe-anti-mouse-fcepsilonrialpha-antibody-5950?GroupID=BLG6716
15. Siglec-f(#155528,Clone-S17007L, Biolgend INC, USA, 1:1000) https://www.biolegend.com/en-us/products/purified-anti-mouse-cd170-siglec-f-antibody-16369
16. IFNγ – AF647 (#505814,Clone-XMG1.2, Biolegend INC, USA, 1:1000) https://www.biolegend.com/fr-fr/products/alexa-fluor-647-anti-mouse-ifn-gamma-antibody-2722
17. IL-17 – PE-cy7 (#506922, Clone-TC11-18H10.1,Biolegend INC, USA, 1:1000) https://www.biolegend.com/en-us/products/pe-cyanine7-anti-mouse-il-17a-antibody-6013?GroupID=GROUP24
18. IL-10 – PE (#505008, Clone-JES5-16E3, Biolegend INC, USA, 5:1000) https://www.biolegend.com/en-us/products/pe-anti-mouse-il-10-antibody-944?GroupID=GROUP24
19. Foxp3 – AF647 (#126408,Clone-MF14, Biolegend, USA,2:1000) https://www.biolegend.com/en-us/products/alexa-fluor-647-anti-mouse-foxp3-antibody-4662
20. IL-9 – Percp-cy5.5 (#514112,Clone-RM9A4, Biolegend INC, USA, 5:1000) https://www.biolegend.com/en-us/clone-search/percp-cyanine5-5-anti-mouse-il-9-antibody-9037
21. IL-4 – PE(#504104, Clone-11B11,Biolegend, USA, 1:1000) https://www.biolegend.com/ja-jp/products/pe-anti-mouse-il-4-antibody-893?GroupID=BLG1753
22. anti-mouse CD3 FITC (# 100204, Clone-17A2, Biolegend INC, USA, 2:2000) https://www.biolegend.com/en-us/products/fitc-anti-mouse-cd3-antibody-45?GroupID=BLG6732
23. anti-mouse CD11b FITC (#101206,Clone-M1/70, Biolegend INC,USA, 1:1000) https://www.biolegend.com/en-us/products/fitc-anti-mouse-human-cd11b-antibody-347?GroupID=BLG10660
24. anti-mouse B220- FITC (#103206,Clone-RA3-6B2, Biolegend INC, USA, 2:2000) https://www.biolegend.com/en-us/products/fitc-anti-mouse-human-cd45r-b220-antibody-445?GroupID=GROUP658
25. IL-9 – APC (#514106, Clone-RM9A4, Biolegend, USA, 3:3000) https://www.biolegend.com/en-us/products/apc-anti-mouse-il-9-antibody-5980?GroupID=GROUP24

Eukaryotic cell lines

Policy information about cell lines and Sex and Gender in Research

Cell line source(s)	VeroE6 (ATCC CRL-1587), A549 (Adenocarcinomic human alveolar basal epithelial cell line; ATCC-CCL185), Caco2 cells (Colon epithelial cell; HTB-37) were kind gift from Dr. Sweety Samal.
Authentication	Authenticated by STR method
Mycoplasma contamination	All cell lines were negative for Mycoplasma contamination.
Commonly misidentified lines (See ICLAC register)	None of the used cell lines is listed in ICLAC database

Animals and other research organisms

Policy information about studies involving animals; ARRIVE guidelines recommended for reporting animal research, and Sex and Gender in Research

Laboratory animals: AB6.Cg-Tg(K18-ACE2)2Prlmn/j mice (strain:034860, Common name: K18-hACE2 mice), Foxo1fl/flxCD4Cre+ (Foxofl/fl:strain#024756; CD4Cre strain:017336), mTmG mice (B6.129(Cg)-Gt(ROSA)26Sortm4(ACTB-tdTomato,-EGFP)Luo/J; strain #007676) were procured from Jackson Laboratory and bred at THSTI. Heterozygous hACE2 transgenic mice, 6-12 weeks old and mixed gender were used for all the experiments. Laboratory animals were housed at institutional animal house Facility maintained between 19 to 26 degrees ambient temperature with 30-70% humidity and 14h light and 10h dark cycle. All animal procedures containing infection were performed in laminor flow hoods inside BSL3 facility.

Wild animals: AB6.Cg-Tg(K18-ACE2)2Prlmn/j mice (strain:034860, Common name: K18-hACE2 mice), Foxo1fl/flxCD4Cre+ (Foxofl/fl:strain#024756; CD4Cre strain:017336), mTmG mice (B6.129(Cg)-Gt(ROSA)26Sortm4(ACTB-tdTomato,-EGFP)Luo/J; strain #007676) were procured from Jackson Laboratory and bred at THSTI. Heterozygous hACE2 transgenic mice, 6-12 weeks old and mixed gender were used for all the experiments. Laboratory animals were housed at institutional animal house Facility maintained between 19 to 26 degrees ambient temperature with 30-70% humidity and 14h light and 10h dark cycle. All animal procedures containing infection were performed in laminor flow hoods inside BSL3 facility.

Reporting on sex: We used both the gender (Male and Female) mice for all the experiments and distributed equally without any biasness.

Field-collected samples: No field collected samples were used in the study.

Ethics oversight: All the experiments were performed at infectious disease research facilility (IDRF) in BSL-3 and ABSL-3 as per IBSC (Institutional Biosafety committee) guidelines. All experimental procedures involving virus challenge were approved by the Institutional Animal Ethics Committee (IAEC), IBSC and RCGM as per the guidelines of THSTI (IAEC/THSTI/191) and Department of Biotechnology, Govt. of India.

Note that full information on the approval of the study protocol must also be provided in the manuscript.

Flow Cytometry

Plots

Confirm that:

- [x] The axis labels state the marker and fluorochrome used (e.g. CD4-FITC).
- [x] The axis scales are clearly visible. Include numbers along axes only for bottom left plot of group (a 'group' is an analysis of identical markers).
- [ ] All plots are contour plots with outliers or pseudocolor plots.
- [x] A numerical value for number of cells or percentage (with statistics) is provided.

Methodology

Sample preparation: Flow cytometry and Intracellular cytokine staining
Processed cells from BALF, dLN or Spleen were stained for surface markers by using FACS antibodies in FACS buffer (PBS with 1% FBS) as previously described (Taemann et al., 1998; Malik S et al., 2017). Briefly, cells were stimulated with prorbol 12-myristate 13-acetate (PMA-50ng/ml; Sigma-Aldrich) for 4-5 h in presence of Monensin (#554724 Golgi-stop, BD Biosciences). Thereafter, cells were washed and incubated with Fc block (anti-mouse CD16/32, Biolegend) at room temperature for 20 min followed by surface staining for 15-20 min at RT in dark and then the cells were fixed in Cytofix and permiabilised with perm/wash buffer using Fixation permiabilisation solution kit (#554714, BD Bioscience). Thereafter, permiabilised cells were used for Intracellular cytoine by using respective antibodies in permiabilisng buffer in dark 1h at 4 degrees celcious. For surface staining only, freshly isolated cells were first blocked with Fc block and then stained directly with desired antibodies. The cell were then washed and analyzed by Flow cytometry (Canto II; BD Biosciences). Data analysis was performed using Flowjo software (Tree-star). For cytokine intra cellular staining cells were stained for surface molecules and then fixated and permiabilised using BD Cytofix/Cyto perm buffer (BD).
All the samples were processed inside the BSL3, after cells were fixed and stained we brought the samples to outside by following IBSC guidlines.

Instrument: FACS Canto (BD Biosciences), BD Facs Aria III (BD), and BD FACS Symphony TM instrument.

Software: FACS Diva software version 8.0.2 (BD), Flowjo software (10 Tree star)

Cell population abundance: BALF cells (30k), Spleen (0.1 million), and dLN (0.1 million were used further for surface, intra cellular cytokine staining to identify the different cell population and cytokine levels.

Gating strategy

Gating strategy for intra cellular staining: Cells were activated and gated on FSC vs SSC, and further gated on live cells. Live cells were further gated on CD4, CD8+ T cells in which intra cellular cytoikne levels were tested.

☒ Tick this box to confirm that a figure exemplifying the gating strategy is provided in the Supplementary Information.
